# Self‐Propelled Proteomotors with Active Cell‐Free mtDNA Clearance for Enhanced Therapy of Sepsis‐Associated Acute Lung Injury

**DOI:** 10.1002/advs.202301635

**Published:** 2023-07-30

**Authors:** Weichang Huang, Lihong Wen, Hao Tian, Jiamiao Jiang, Meihuan Liu, Yicheng Ye, Junbin Gao, Ruotian Zhang, Fei Wang, Huaan Li, Lihan Shen, Fei Peng, Yingfeng Tu

**Affiliations:** ^1^ Department of Critical Care Medicine Dongguan Institute of Respiratory and Critical Care Medicine Affiliated Dongguan Hospital Southern Medical University Dongguan 523059 China; ^2^ NMPA Key Laboratory for Research and Evaluation of Drug Metabolism & Guangdong Provincial Key Laboratory of New Drug Screening School of Pharmaceutical Sciences Southern Medical University Guangzhou 510515 China; ^3^ Department of Plastic Surgery Sun Yat‐sen Memorial Hospital Sun Yat‐sen University Guangzhou 510120 China; ^4^ School of Materials Science and Engineering Sun Yat‐Sen University Guangzhou 510275 China

**Keywords:** cell‐free mitochondrial DNA, DNase‐I, nanomotors, pulmonary delivery, self‐propulsion

## Abstract

Acute lung injury (ALI) is a frequent and serious complication of sepsis with limited therapeutic options. Gaining insights into the inflammatory dysregulation that causes sepsis‐associated ALI can help develop new therapeutic strategies. Herein, the crucial role of cell‐free mitochondrial DNA (cf‐mtDNA) in the regulation of alveolar macrophage activation during sepsis‐associated ALI is identified. Most importantly, a biocompatible hybrid protein nanomotor (NM) composed of recombinant deoxyribonuclease I (DNase‐I) and human serum albumin (HSA) via glutaraldehyde‐mediated crosslinking is prepared to obtain an inhalable nanotherapeutic platform targeting pulmonary cf‐mtDNA clearance. The synthesized DNase‐I/HSA NMs are endowed with self‐propulsive capability and demonstrate superior performances in stability, DNA hydrolysis, and biosafety. Pulmonary delivery of DNase‐I/HSA NMs effectively eliminates cf‐mtDNAs in the lungs, and also improves sepsis survival by attenuating pulmonary inflammation and lung injury. Therefore, pulmonary cf‐mtDNA clearance strategy using DNase‐I/HSA NMs is considered to be an attractive approach for sepsis‐associated ALI.

## Graphical abstract

A biocompatible hybrid protein nanomotor (NM) composed of recombinant deoxyribonuclease I (DNase‐I) and human serum albumin (HSA) is prepared via glutaraldehyde‐mediated crosslinking, which exhibits enhanced diffusion by catalyzing cf‐DNA hydrolysis. After pulmonary delivery, DNase‐I/HSA NMs effectively block cf‐mtDNA/TLR9‐mediated alveolar macrophage activation by efficiently scavenging cf‐mtDNA in the pulmonary microenvironment, thereby alleviating sepsis‐associated acute lung injury.

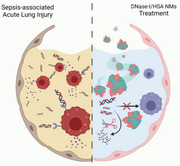

## 1 Introduction

Sepsis is a devastating clinical disorder response to infection that involves aberrant immune response and dysregulated inflammatory reaction, leading to multiple organ failure and death.^[^ ^1^ ^]^ Despite significant attempts to improve its therapeutic treatment, sepsis remains a major concern globally and results in more than 11 million fatalities annually.^[^ ^2^ ^]^ Notably, the lung is one of the most vulnerable organs during sepsis, which clinically manifests as acute lung injury (ALI).^[^ ^3^ ^]^ ALI represents the major devastating complication of sepsis that contributes to higher mortality rates and poorer prognoses among septic patients, despite the progresses of mechanical ventilation and symptomatic treatment.^[^ ^4^ ^]^ Hence, it is of great significance to develop novel and potential therapeutic approaches for the management of sepsis‐associated ALI.

In sepsis, the dysregulated immunoinflammatory response is typically triggered and driven by the overactivation of pattern recognition receptors, which recognize danger signals such as pathogen‐associated molecular patterns or endogenous damage‐associated molecular patterns.^[^ ^5^, ^6^ ^]^ Among those danger signals, cell‐free DNAs (cf‐DNAs), including damaged cell‐released nuclear DNA and mitochondrial DNA, as well as neutrophil extracellular traps, have been identified to not only serve as the prognostic and predictive biomarker of sepsis but also contribute to organ damage and mortality in sepsis.^[^ ^7^, ^8^, ^9^, ^10^ ^]^ It has been demonstrated that cf‐DNAs released from the damaged cells are possible to activate Toll‐like receptor 9 (TLR9)‐mediated proinflammatory signaling in innate immune cells such as resident macrophages and contribute to the degree and duration of inflammation.^[^ ^11^, ^12^ ^]^ Of note, within the cf‐DNAs fraction, cell‐free mitochondrial DNA (cf‐mtDNA), which is phylogenetically derived from bacteria and also rich in hypomethylated CpG sequences, is considered more prone to induce inflammation than cell‐free nuclear DNA (cf‐nDNA).^[^ ^11^, ^13^, ^14^ ^]^ Moreover, the accumulation of cf‐DNAs has been observed in local damaged tissues in inflammation‐related diseases, including rheumatoid arthritis,^[^ ^15^ ^]^ psoriasis,^[^ ^16^ ^]^ and inflammatory bowel disease,^[^ ^17^ ^]^ which served as a pivotal regulator in mediating tissue damage and disease progression. Most importantly, targeting the clearance of accumulated cf‐DNAs in local damaged tissues has been proved to be an attractive treatment strategy for these diseases. Recently, various clinical studies have shown that elevated plasma cf‐DNAs levels, particularly cf‐mtDNA levels, are related to an increased rate of deterioration in sepsis‐associated lung injury.^[^ ^15^, ^16^, ^17^ ^]^ However, still very little is known about the variation of cf‐DNAs content in lung tissues during sepsis‐associated ALI and their association with disease progression, which needs further exploration.

Pulmonary delivery of recombinant deoxyribonuclease I (DNase‐I) can effectively remove the extracellular DNA in the airways of cystic fibrosis patients, which results in clear mucus and ameliorated symptoms.^[^ ^18^ ^]^ Recently, clinical trials have suggested that degradation of localized extracellular DNA in lungs by inhaled DNase‐I can be beneficial for acute respiratory failure in COVID‐19.^[^ ^19^, ^20^, ^21^ ^]^ However, the positive effects were limited to the time of drug retention,^[^ ^21^ ^]^ presumably due to rapid clearance of DNase‐I from the lung.^[^ ^22^, ^23^ ^]^ The longer lung retention was accompanied by improved therapeutic efficacy for pulmonary drug delivery.^[^ ^24^, ^25^ ^]^ In recent years, biomedical nanotechnology has received extensive attention in improving drug delivery efficiency,^[^ ^26^ ^]^ which has a broad clinic application prospect in sepsis therapy.^[^ ^27^, ^28^ ^]^ The properties of nanoparticles, such as shape, size, and surface charge, help in their longer half‐life and targeted delivery, in comparison to the conventional formulations.^[^ ^29^, ^30^, ^31^ ^]^ Previous studies have demonstrated that immobilization of DNase‐I on the nanoparticle surface effectively improves the stability and prolongs biological half‐life.^[^ ^32^, ^33^ ^]^ Of note, nanomotors (NMs), the self‐propelled nanoparticles capable of utilizing surrounding energies (chemical or physical energies) to generate mechanical motion, have attracted considerable attention for biomedical applications,^[^ ^34^, ^35^ ^]^ including precise drug delivery, diagnosis, bio‐sensing, and noninvasive surgery. Most importantly, the motion capability of NMs can substantially accelerate the interaction between the target molecules and NMs,^[^ ^36^ ^]^ which greatly enhances the capture performance^[^ ^37^ ^]^ or catalytic efficiency.^[^ ^38^ ^]^ Enzymes are proteins‐based biocatalysts with high catalytic efficiency, which exhibit enhanced diffusion in presence of substrates.^[^ ^39^ ^]^ Moreover, enzymes can also be incorporated into artificially synthesized nanoarchitectures and propel these nanostructures via catalytic reaction, which have been classified as enzyme‐powered NMs.^[^ ^40^, ^41^, ^42^ ^]^

**Figure 1:**
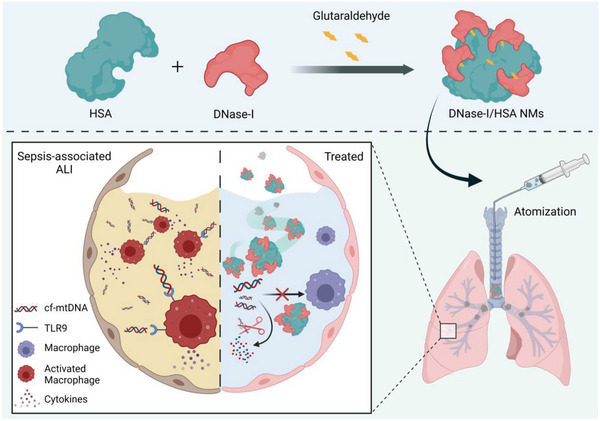
The synthetic procedure of DNase‐I/HSA NMs and their application for sepsis‐associated ALI treating. Fabrication of DNase‐I/HSA NMs via glutaraldehyde‐mediated crosslinking of HSA and DNase‐I (upper panel). After aerosolized intratracheal instillation, self‐propulsive DNase‐I/HSA NMs effectively blocked cf‐mtDNA/TLR9‐mediated alveolar macrophage activation by efficiently scavenging cf‐mtDNA in the pulmonary microenvironment, thereby alleviating sepsis‐associated ALI (lower panel).

In this study, we sought to determine if cf‐DNAs in lung tissues were accumulated during sepsis‐associated ALI and whether eliminating local cf‐DNAs could be used as an effective therapeutic approach for sepsis‐associated ALI. We analyzed the local cf‐DNAs levels in the lungs of mice with sepsis‐associated ALI to determine the association of pulmonary cf‐DNAs with the innate immune response and lung injury during sepsis. Furthermore, we prepared hybrid protein NMs via glutaraldehyde‐mediated crosslinking of human serum albumin (HSA), the most abundant protein in plasma,^[^ ^43^ ^]^ and DNase‐I, the recombinant enzyme efficiently hydrolyzing DNA fragments, at an optimized molar ratio. The obtained DNase‐I/HSA NMs demonstrated excellent properties, for instance, outstanding physiological stability, well‐retained enzymatic activity, as well as favorable biocompatibility. Notably, DNase‐I/HSA NMs exhibited enhanced diffusion even under a low concentration of dsDNAs fuel, due to the efficient catalytic capacity of DNase‐I. Additionally, owing to the enhanced diffusion and appropriate particle size, the retention time of DNase‐I/HSA NMs in lung tissues was significantly extended. Moreover, pulmonary delivery of DNase‐I/HSA NMs remarkably eliminated cf‐mtDNA in the lungs of mice with sepsis‐associated ALI and also effectively improved sepsis survival by attenuating pulmonary inflammation and lung injury, as illustrated in **Figure** **1**. Together, this work highlights the potential of scavenging pulmonary cf‐mtDNA via DNase‐I/HSA NMs for the treatment of sepsis‐associated ALI.

## 2 Results and Discussion

### 2.1 Increased cf‐mtDNA Level in Lung Tissues was Associated with Inflammation in Sepsis‐Associated ALI

**Figure 2:**
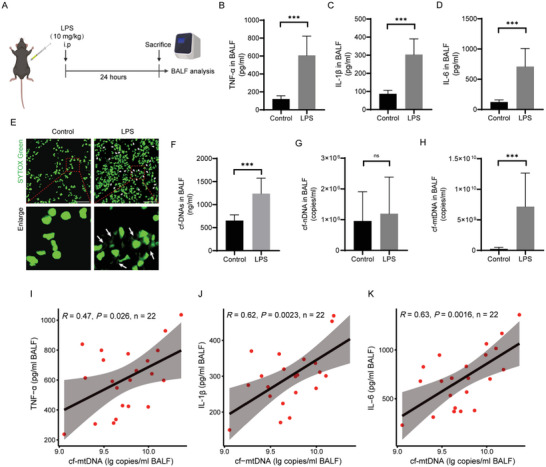
Increased cf‐mtDNA level was associated with inflammation in sepsis‐associated ALI. A) Schematic representation of the experiment schedule. Sepsis‐associated ALI model was induced by LPS, and BALF was collected at 24 h for further analysis. B–D) The BALF levels of TNF‐α, IL‐1β, and IL‐6 in the septic mice (*n* = 22) and healthy subjects (*n* = 10) were determined by enzyme‐linked immunosorbent assay (ELISA). E) Confocal images of cf‐DNAs in lung tissues of mice stained with SYTOX Green (scale bar: 50 µm). Enlarged images showed the intertwined strand structure of cf‐DNAs (white arrow). F) cf‐DNAs level in BALF of septic mice (*n* = 22) and healthy subjects (*n* = 10) was measured using the dsDNA HS assay kit. G,H) Levels of G) cf‐nDNA and H) cf‐mtDNA in the BALF of septic mice (*n* = 22) and healthy subjects (*n* = 10) were evaluated by RT‐qPCR. I–K) Correlation of fold‐change between cf‐mtDNA and I) TNF‐α, J) IL‐1β, and K) IL‐6 in the BALF from septic mice (*n* = 22) (Spearman's correlation test for cf‐mtDNA). Data represent mean ± SD; differences were compared by Student's *t*‐test. *p < 0.05, **p < 0.01, ***p < 0.001; ns, no significant difference.

As is well known, cf‐DNAs can activate DNA sensors in immune cells, resulting in increased expression of multiple inflammatory cytokines, such as TNF‐α, IL‐1β, and IL‐6,^[^ ^44^, ^45^ ^]^ which are associated with the progression of sepsis‐associated ALI.^[^ ^46^ ^]^ To further clarify the relationship between cf‐DNAs and inflammation in sepsis‐associated ALI, we constructed a mouse model of sepsis‐associated ALI with the intraperitoneal administration of Lipopolysaccharides (LPS) (10 mg kg^−1^) (**Figure** **2**A).^[^ ^47^ ^]^ We evaluated the changes in the expression of inflammatory cytokines in bronchoalveolar lavage fluid (BALF) and found that the levels of TNF‐α, IL‐1β, and IL‐6 in the BALF of septic mice were significantly higher than those in the healthy controls (Figure 2B–D), indicating that the sepsis‐associated ALI mouse model was constructed successfully.^[^ ^48^ ^]^ To detect cf‐DNAs in lung tissues, the frozen tissue sections were stained with SYTOX Green, a nucleic acid‐specific, green‐fluorescent dye, which is intense positive for extracellular DNA.^[^ ^49^ ^]^ These extracellular DNAs, also known as cf‐DNAs, could be distinguished from nuclear chromosomes by the structure of tangled strands and the appearance of barbed wire.^[^ ^49^ ^]^ In lung sections of septic mice, such cf‐DNAs with an intertwined strand structure were significantly increased as compared to the healthy control (Figure 2E, indicated by arrows). Consistently, the BALF level of cf‐DNAs was modestly increased from 0.65 to 1.24 µg mL^−1^ during sepsis‐associated ALI (Figure 2F). Furthermore, we separately measured the levels of cf‐nDNA and cf‐mtDNA, two main components of cf‐DNAs, in BALF using quantification real‐time polymerase chain reaction (RT‐PCR). Interestingly, the cf‐mtDNA level in BALF was substantially elevated during sepsis‐associated ALI, but the level of cf‐nDNA was shown not to vary significantly (Figure 2G,H). Meanwhile, we determined that the BALF level of cf‐mtDNA in septic mice obviously increased after 6 h, peaked at 24 h, and then slightly declined at 48 h, while the changes in cf‐nDNA were less prominent (Figure S1A,B, Supporting Information). Notably, the BALF level of cf‐mtDNA in septic mice was positively correlated with the BALF levels of TNF‐α, IL‐1β, and IL‐6 (Figure 2I–K). Together, these results suggest that the BALF cf‐mtDNA level, but not BALF cf‐nDNA level, was substantially increased during sepsis‐associated ALI, which might contribute to the abnormal inflammation.

### 2.2 Pulmonary cf‐mtDNA Aggravated Sepsis‐Associated ALI by Activating Alveolar Macrophages

**Figure 3:**
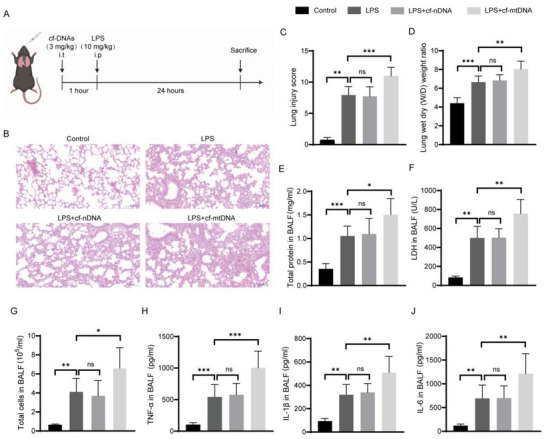
Pulmonary cf‐mtDNA contributed to sepsis‐associated ALI by activating alveolar macrophages. A) Schematic representation of the experiment schedule. cf‐nDNA and cf‐mtDNA were, respectively, administered through aerosolized intratracheal instillation to mice 1 h before intraperitoneal LPS treatment. B) Representative images of lung sections stained with hematoxylin‐eosin (HE, scale bar: 100 µm). C) The corresponding lung injury scores were determined and calculated according to the established criteria (*n* = 6). D,E) Lung tissue edema was measured by D) lung W/D weight ratio and E) BALF protein content (*n* = 6). F) LDH activity in BALF was determined by an LDH cytotoxicity assay kit (*n* = 5). G) Total number of inflammatory cells in BALF were counted via a hemocytometer (*n* = 6). H–J) The BALF levels of TNF‐α, IL‐1β, and IL‐6 were determined by ELISA (*n* = 8). Data represent mean ± SD; differences were compared by one‐way analysis of variance (ANOVA) with Tukey's multiple comparisons test. *p < 0.05, **p < 0.01, ***p < 0.001; ns, no significant difference.

To further verify the effects of local cf‐DNAs in sepsis‐associated ALI, we, respectively, administered cf‐nDNA and cf‐mtDNA through aerosolized intratracheal instillation to mice 1 h before intraperitoneal LPS treatment, then lung injury and inflammation were assessed 24 h after sepsis (**Figure** **3**A). As expected, LPS stimulation resulted in ALI as revealed by morphological damage (Figure 3B,C), pulmonary edema (Figure 3D,E), as well as elevated lactate dehydrogenase (LDH) in BALF (Figure 3F). Notably, we found that the lung injury was significantly aggravated in the mice that received combined treatment with cf‐mtDNA and LPS compared with LPS alone (Figure 3B–F). Additionally, we also observed a dramatic increase in inflammatory cells infiltration (Figure 3G) and proinflammatory cytokines secretion, including TNF‐α (Figure 3H), IL‐1β (Figure 3I) as well as IL‐6 (Figure 3J), in the BALF of LPS‐induced mice compared with those of controls. Similarly, treatment of cf‐mtDNA together with LPS promoted inflammatory cells infiltration (Figure 3G) and increased production of proinflammatory cytokines (Figure 3H–J). However, pretreatment with cf‐nDNA did not induce noteworthy changes in lung injury and inflammation during sepsis (Figure 3B–J). Thus, our findings had revealed that cf‐mtDNA, rather than cf‐nDNA, was an important endogenous harmful molecule provoking the pulmonary inflammation and lung injury during sepsis, and extended previous researches suggesting the association of excessive pulmonary cf‐mtDNA with disease severity of asthma,^[^ ^50^ ^]^ chronic obstructive pulmonary disease,^[^ ^51^ ^]^ as well as idiopathic pulmonary fibrosis.^[^ ^52^ ^]^ According to the theory of mitochondrial origin,^[^ ^53^ ^]^ mitochondria evolved from endosymbiotic proteobacteria. Similar to bacterial DNA, mtDNA contains a significant amount of unmethylated CpG dinucleotides, which could be recognized by the innate immune system.^[^ ^54^ ^]^

Alveolar macrophage plays an essential role in the pathogenesis of sepsis‐associated ALI.^[^ ^3^ ^]^ It is well known that cf‐DNAs mainly exert immunostimulatory effects via the activation of TLR9.^[^ ^12^ ^]^ Thus, we examined the role of cf‐DNAs in alveolar macrophage activation during sepsis‐associated ALI. After co‐incubation with BALF obtained from septic mice for 6 h, the mRNA levels of proinflammatory cytokines (Figure S2A–C, Supporting Information), as well as the expressions of TLR9 (Figure S2D, Supporting Information) and iNOS (Figure S2E, Supporting Information) were markedly increased in alveolar macrophage cells (MH‐S), suggesting TLR9‐mediated activation of macrophages. However, these effects were significantly attenuated by DNase‐I treatment (Figure S2A–E, Supporting Information). These findings implied that pulmonary cf‐DNAs played a critical role in alveolar macrophage activation during sepsis. Furthermore, cf‐nDNA and cf‐mtDNA were used to stimulate MH‐S cells for 6 h, respectively. As expected, alveolar macrophages were effectively activated upon cf‐mtDNA stimulation, manifested as increased mRNA levels of proinflammatory cytokines (Figure S3A–C, Supporting Information). Consistent with previous observations, cf‐nDNA failed to activate alveolar macrophages (Figure S3A–C, Supporting Information).

Taken together, these results confirmed that pulmonary cf‐mtDNA, but not cf‐nDNA, played a crucial role in sepsis‐associated ALI by promoting the activation of alveolar macrophage. Thus, targeting pulmonary cf‐mtDNA might be an effective strategy for sepsis‐associated ALI.

### 2.3 Preparation and Characterization of DNase‐I/HSA NMs

**Figure 4:**
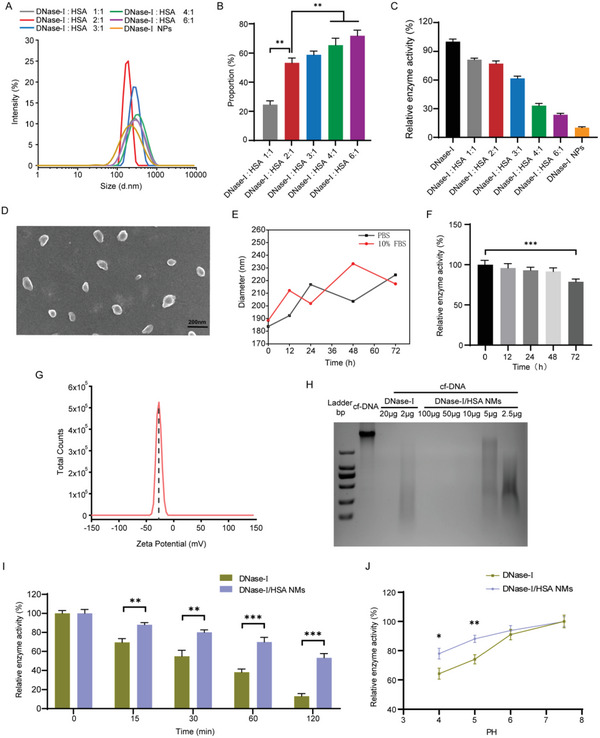
Characterization of DNase‐I/HSA NMs. A) Hydrodynamic size of DNase‐I/HSA NMs was confirmed by DLS. B) The proportion of DNase‐I in the form of DNase‐I/HSA NMs was measured by fluorescence spectrophotometry (*n* = 5). C) Enzymatic activity of free DNase‐I and DNase‐I/HSA NMs measured by fluorometric DNase assay kit (*n* = 6). D) SEM image of DNase‐I/HSA NMs (scale bar: 200 nm). E) Hydrodynamic diameter evolution profiles of DNase‐I/HSA NMs incubated in PBS only or PBS containing 10% FBS at room temperature for 72 h. F) Enzymatic activity evolution profiles of DNase‐I/HSA NMs incubated in PBS containing 10% FBS at room temperature for 72 h (*n* = 3). G) Surface charge of DNase‐I/HSA NMs measured by electrophoretic mobility analysis. H) DNA digestion capability of free DNase‐I and DNase‐I/HSA NMs measured by agarose gel electrophoresis. I) Relative enzymatic activities of free DNase‐I and DNase‐I/HSA NMs after incubation with protease K for different time (*n* = 3). J) Relative enzymatic activities of free DNase‐I and DNase‐I/HSA NMs after being incubated at different pH for 2 h (*n* = 3). All experiments were performed at least in triplicate. Data represent mean ± SD; differences were compared by Student's *t*‐test or one‐way ANOVA with Tukey's multiple comparisons test. *p < 0.05, **p < 0.01, ***p < 0.001.

DNase‐I is an endonuclease that nonspecifically cleaves DNA fragments, and has been approved for the treatment of cystic fibrosis.^[^ ^18^ ^]^ Inhaled DNase‐I can effectively remove extracellular DNAs in the respiratory tract, however, its rapid elimination from the lung is a considerable drawback to its full clinical benefit.^[^ ^22^ ^]^ Thus, we prepared DNase‐I/HSA NMs by crosslinking HSA and DNase‐I using glutaraldehyde as the crosslinker to serve as long‐acting DNase‐I, since protein hybridization can enhance the stability of enzyme and thereby prolonging residence time.^[^ ^43^ ^]^ In order to screen for the optimal molar ratio, we tested five groups of DNase‐I/HSA hybrid proteins (DNase‐I/HSA molar ratio at 1:1, 2:1, 3:1, 4:1, to 6:1, respectively). Based on dynamic light scattering (DLS) analysis, the synthesized DNase‐I/HSA NMs at the molar ratio of 2:1 showed an average hydrodynamic diameter of around 190 nm (**Figure** **4**A), with a uniform size distribution (polydispersity index: 0.17) (Figure S4A, Supporting Information). Moreover, the particle size of the hybrid proteins with other molar ratios is relatively large (about 300 nm) and therefore unsuitable for pulmonary delivery. In addition, the proportion of DNase‐I in NMs was also increased when we increased the molar ratio of DNase‐I (Figure 4B). However, it resulted in a significant decrease in the enzymatic activity (Figure 4C), which might be attributed to the masking of the active site of DNase‐I during the crosslinking process. Notably, DNase‐I nanoparticles (NPs) synthesized via self‐crosslinking had an average hydrodynamic size of 230 nm (Figure 4A), but the enzyme activity was obviously reduced by about 90% (Figure 4C). When the molar ratio of DNase‐I to HSA was 2:1, the synthesized DNase‐I/HSA NMs exhibited a higher proportion of DNase‐I (53.30%) (Figure 4B) and better enzyme activity (77.23%) (Figure 4C), as well as the ideal particle size. Therefore, a 2:1 molar ratio of DNase‐I to HSA was chosen for the following study.

According to scanning electron microscope (SEM, Figure 4D) and transmission electron microscope (TEM, Figure S4B, Supporting Information) observations, DNase‐I/HSA NMs exhibited an incomplete symmetric spherical structure with an average diameter of ≈153.6 nm and were well dispersed. Most interestingly, during being incubated in either phosphate‐buffered saline (PBS) or PBS with 10% fetal bovine serum (FBS), such DNase‐I/HSA NMs exhibited the modest fluctuation in particle size (Figure 4E) and a slight decrease (about 20%) in enzyme activity (Figure 4F) within 72 h, demonstrating the favorable stability. Additionally, the surface charge of DNase‐I/HSA NMs was measured to be −27.1 mV, which might contribute to the excellent dispersion stability (Figure 4G).^[^ ^55^ ^]^ The DNA degradation capability of DNase‐I/HSA NMs was also evaluated via agarose gel electrophoresis. In accordance with the fluorometric DNase assay (Figure 4C), the catalytic capacity of such DNase‐I/HSA NM was ≈75% of that of the free DNase‐I (Figure 4H), suggesting that the enzymatic activity of DNase‐I/HSA NMs was well retained. Moreover, considering that protease widely exists in the biological environment and plays an essential role in the degradation of enzymes,^[^ ^56^ ^]^ we then evaluated the resistance of such DNase‐I/HSA NMs toward protease K digestion.^[^ ^43^ ^]^ Following protease K challenge (0.4 mg mL^−1^) for 2 h, such DNase‐I/HSA NMs retained about 53% of enzymatic capacity as assessed by fluorometric DNase assay kit, whereas free DNase‐I exhibited only 13% (Figure 4I). pH is one of the important factors affecting enzyme activity. As the inflammatory microenvironment is known to be acidic,^[^ ^57^ ^]^ we further investigated the catalytic stability of such DNase‐I/HSA NMs at different acidic pH (4.0, 5.0, 6.0, and 7.5), which might characterize the inflammatory microenvironment local pH (3.6–5.7). Unlike free DNase‐I that the enzyme activity was obviously diminished after 2 h of incubation in buffers of pH 4.0 or 5.0, DNase‐I/HSA NMs still preserved higher catalytic activity following challenge (Figure 4J).

Overall, these results indicated that such DNase‐I/HSA NMs exhibited excellent stability in extreme conditions, making them more suitable for therapeutic applications.

### 2.4 Enhanced Diffusion of DNase‐I/HSA NMs

**Figure 5:**
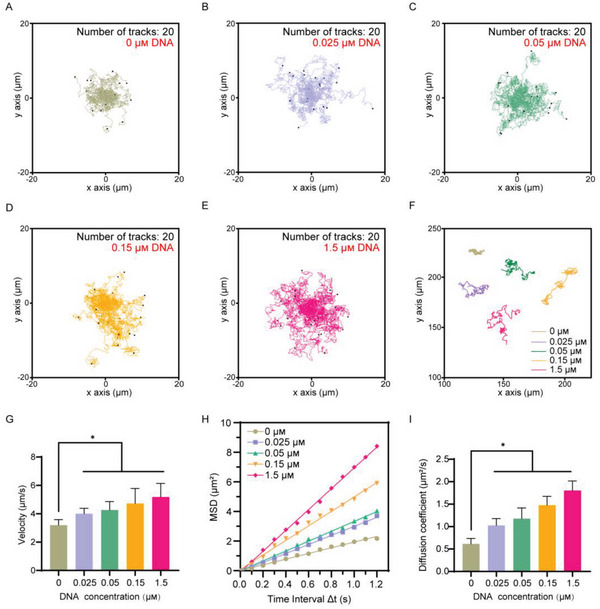
Motion analysis of DNase‐I/HSA NMs. A–E) Enhanced diffusion of DNase‐I/HSA NMs in the presence of different dsDNA concentrations: A) 0 × 10^−6^ m, B) 0.025 × 10^−6^ m, C) 0.05 × 10^−6^ m, D) 0.15 × 10^−6^ m, and E) 1.5 × 10^−6^ m. F) Typical tracking trajectories of DNase‐I/HSA NMs. G) The average velocity of DNase‐I/HSA NMs. H) MSD analysis of DNase‐I/HSA NMs. I) Diffusion coefficient of DNase‐I/HSA NMs measured by DLS. The motion of DNase‐I/HSA NMs was analyzed with Image J for 20 s (*n* = 20). Data represent mean ± SD; differences were compared by one‐way ANOVA with Tukey's multiple comparisons test. *p < 0.05.

Typically, a geometric asymmetry is constructed on the micro‐/nanomotors (e.g., Janus particles) to achieve an asymmetrical generation of forces,^[^ ^58^ ^]^ which is the critical factor required for self‐propulsion at low Reynolds numbers. Recently, increasing studies have demonstrated that NMs with symmetric structure can also be powered by enzyme catalysis,^[^ ^41^, ^59^ ^]^ which may be attributed to the asymmetric distribution of enzyme molecules around the micro/nanomotors.^[^ ^60^ ^]^ Our previous study has shown that, DNase‐I‐functionalized NMs could generate enhanced diffusion driven by self‐diffusiophoresis in the presence of substrate dsDNA.^[^ ^42^ ^]^ Thus, we carefully investigated the motion profiles of DNase‐I/HSA NMs. The autonomous movement of DNase‐I/HSA NMs was recorded by a Nikon Ti2‐A inverted optical microscope under different concentrations of dsDNA in Dulbecco's PBS (DPBS, with Ca^2+^ and Mg^2+^). Image J with manual tracking module was then used to further analyze the tracking images. Above all, the enhanced diffusion of DNase‐I/HSA NMs was observed in the presence of dsDNA with a concentration‐dependent manner, whereas a typical Brownian motion was recorded under dsDNA‐free condition (**Figure** **5**A–E and Movies S1–S5, Supporting Information). Then, typical optical tracking trajectories of DNase‐I/HSA NMs under different concentrations of dsDNA were plotted (Figure 5F). Of note, it has been reported that the investigated compound in BALF was typically diluted at least 20‐ to 40‐ fold comparing to alveolar lining fluid,^[^ ^61^ ^]^ thus the content of cf‐DNAs in pulmonary microenvironment during sepsis‐associated ALI was estimated as about 25–50 µg mL^−1^ according to the BALF data described above (Figure 2F). Interestingly, the average velocity of DNase‐I/HSA NMs reached 5.2 µm s^−1^ upon the addition of 1.5 × 10^−6^ m dsDNA (30 µg mL^−1^), which was 1.6 times higher than that without dsDNA fuel (Figure 5G). Moreover, the moving performance of DNase‐I/HSA NMs was assessed by the mean‐square displacement (MSD) analysis (Figure 5H). As expected, the fitting curve had shown that the MSD value increased along with the higher fuel concentration, indicating enhanced diffusion of DNase‐I/HSA NMs. Furthermore, the diffusion coefficient of DNase‐I/HSA NMs was further measured by DLS under different concentrations of dsDNA. Diffusion coefficient data also showed that DNase‐I/HSA NMs exhibited enhanced diffusion in a concentration‐dependent manner (Figure 5I). Obviously, the diffusion coefficient was 0.6 µm^2^ s^−1^ in the absence of substrate, while it was elevated to 1.8 µm^2^ s^−1^, threefold increase, upon addition of 1.5 × 10^−6^ m dsDNA. Overall, these data suggested that such DNase‐I/HSA NMs exhibit enhanced diffusion in dsDNA‐presented microenvironment, particularly at the pathological level of cf‐DNAs in pulmonary microenvironment during sepsis‐associated ALI, which holds great potential for further biomedical applications.

### 2.5 DNase‐I/HSA NMs Exhibit Long Residence Time in the Lungs

**Figure 6:**
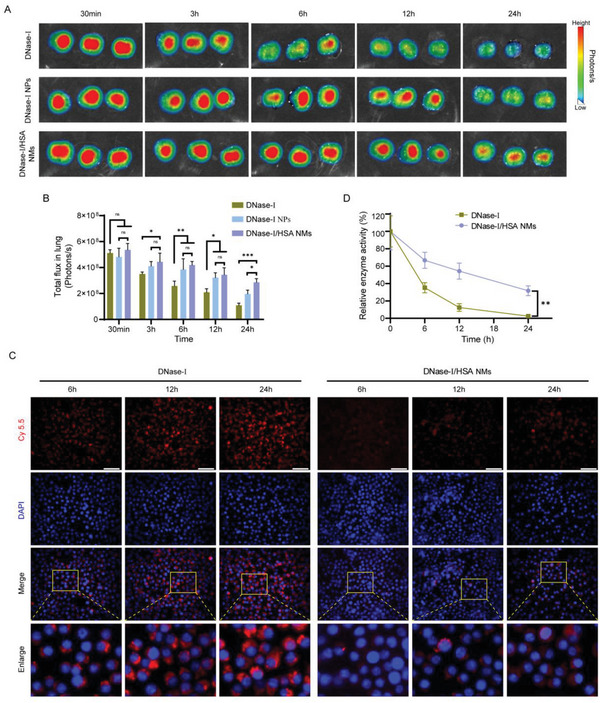
DNase‐I/HSA NMs significantly prolonged the pulmonary retention time compared with free DNase‐I. A) Ex vivo fluorescent imaging of lungs after intratracheal administration at various time points. *n* = 3. B) Signal quantification ROIs in (A). C) In vitro uptake of free DNase‐I and DNase‐I/HSA NMs by alveolar macrophages was observed using florescent microscopy (scale bar: 50 µm). All cell experiments were performed at least in triplicate. D) Enzymatic activity of DNase‐I in the lung homogenates. *n* = 6. Data represent mean ± SD; differences were compared by ANOVA with Tukey's multiple comparisons test. *p < 0.05, **p < 0.01, ***p < 0.001; ns, no significant difference.

To our knowledge, inhalation of therapeutic protein for pulmonary diseases poses a major challenge in clinic due to the rapid elimination from the lungs.^[^ ^62^ ^]^ DNase‐I, a 37 kDa glycoprotein which can effectively remove extracellular DNAs in the respiratory tract following pulmonary delivery,^[^ ^22^ ^]^ is the potential drug for treating sepsis‐associated ALI. However, the rapid clearance of free DNase‐I from the lung is a considerable drawback to its full clinical benefit: when the dose of 2.5 mg is inhaled, a concentration of 3 µg mL^−1^ is measured in sputum immediately after inhalation and it is reduced to 0.6 µg mL^−1^ after 2 h.^[^ ^56^ ^]^ Notably, nanoparticle‐based pulmonary delivery of therapeutic proteins has been proved to be a promising strategy for improving protein bioavailability and prolonging residence time within lung tissues.^[^ ^63^, ^64^, ^65^ ^]^ Thus, the residence time of such DNase‐I/HSA NMs in lung tissues was carefully analyzed. To this end, the fluorescence signal in lung tissues was followed overtime after the intratracheal instillation of indocyanine green (ICG)‐labeled DNase‐I, DNase‐I NPs, or DNase‐I/HSA NMs in mice upon LPS challenge, and the decay of fluorescence intensity over time was used to calculate the elimination half‐life. We determined that the pulmonary elimination half‐life of free DNase‐I was estimated to be about 6 h, whereas that of either DNase‐I NPs or DNase‐I/HSA NMs was extended to be 12–24 h (**Figure** **6**A,B). DNase‐I NPs and DNase‐I/HSA NMs had similar particle sizes but different motility. The diffusion coefficient of DNase‐I NPs in the presence of 1.5 × 10^−6^ m dsDNA is much lower than that of DNase‐I/HSA NMs (Figure S5, Supporting Information). Interestingly, the lung retention of DNase‐I/HSA NMs was moderately higher than that of DNase‐I NPs 24 h after administration (Figure 6A,B), suggesting that the enhanced diffusion of DNase‐I/HSA NMs could effectively improve the lung retention efficiency. It is well known that mucociliary‐dependent clearance is a critical hurdle for pulmonary drug delivery,^[^ ^66^ ^]^ and promoting the mucus penetration of inhaled drug has been considered to be an efficient strategy to evade mucociliary clearance and thus prolong lung retention.^[^ ^67^ ^]^ Recently, micro/nanomotors have been reported to effectively enhance mucus penetration and prolong retention time in target location due to the autonomous motion.^[^ ^68^, ^69^ ^]^ Thus, the enhanced diffusion of DNase‐I/HSA NMs might be an important factor for extending lung retention. Additionally, the uptake by alveolar macrophages was considered as another critical mechanism for pulmonary protein clearance.^[^ ^70^ ^]^ Particles with the diameter between 50 and 200 nm could theoretically escape the detection from alveolar macrophage,^[^ ^63^ ^]^ while DNase‐I/HSA NMs conformed well to this profile. Subsequently, the uptake of free DNase‐I and DNase‐I/HSA NMs across murine alveolar macrophages (MH‐S) was determined by fluorescence microscope. As expected, the uptake of free DNase‐I by alveolar macrophages progressively increases over time, while the uptake was remarkably lower for DNase‐I/HSA NMs (Figure 6C).

Furthermore, we assessed the changes of DNase‐I enzymatic activity in lung tissues following intratracheal administration of free DNase‐I and DNase‐I/HSA NMs, respectively. As shown in Figure 6D, at 24 h post‐delivery, the catalytic activity of DNase‐I in the lung homogenates was significantly decreased compared with the initial measurements and that of DNase‐I/HSA NMs was 13‐fold higher than free DNase‐I. Normally, the enzymatic activity of enzymes in lung tissues is affected by several factors, including protease digestion, local pH influence, and most importantly, rapid clearance from the lungs. Previous studies have shown that crosslinking enzymes with proteins could effectively improve the biological stability of enzymes.^[^ ^43^, ^71^ ^]^ By crosslinking DNase‐I with HAS, our hybrid protein NMs could significantly improve the catalytic stability of DNase‐I during protease or pH challenge (Figure 4I,J). Moreover, DNase‐I/HSA NMs significantly extended the lung retention time of DNase‐I (Figure 6A,B), which also contributed to the reserved catalytic activity of DNase‐I in lung tissues following pulmonary administration.

Collectively, these results demonstrated that such DNase‐I/HSA NMs significantly enhanced lung retention following pulmonary administration, which could effectively improve the bioavailability of DNase‐I.

### 2.6 Enhanced Therapeutic Effect of DNase‐I/HSA NMs in Sepsis‐Associated ALI

**Figure 7:**
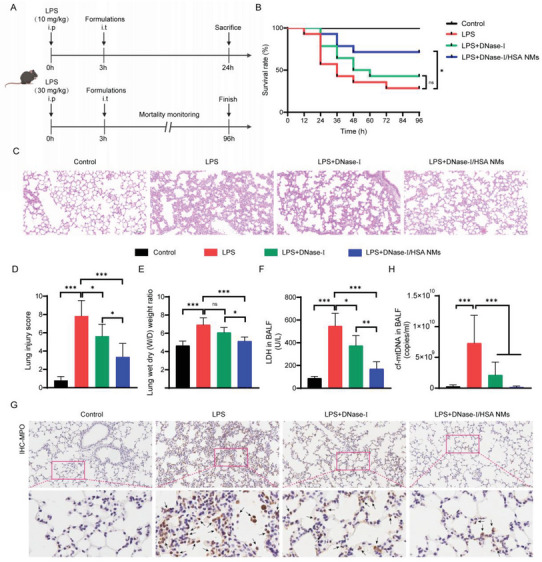
DNase‐I/HSA NMs markedly ameliorated sepsis‐associated ALI. A) Schematic representation of the experiment schedule. Sepsis‐associated ALI mouse model was generated by intraperitoneal administration (i.p) of LPS. 3 h after LPS challenge, mice were intratracheally infused (i.t) with PBS, free DNase‐I, or DNase‐I/HSA NMs. For the therapeutic analysis, the mice were sacrificed at 24 h after LPS (10 mg kg^−1^) challenge (upper panel), or observed every 12 h up to 96 h after challenge with the lethal dose of LPS (30 mg kg^−1^) to monitor the mortality (lower panel). B) Mice survival rate (*n* = 10–15). C) Representative images of HE‐stained lung sections 24 h after LPS stimulation (scale bar: 100 µm). D) Histogram of lung injury scores (*n* = 6). E) The edema degree of lung tissue was calculated using W/D weight ratio (*n* = 6). F) LDH activity in BALF was determined by an LDH cytotoxicity assay kit (*n* = 5). G) Pulmonary neutrophil infiltration was detected by IHC staining for myeloperoxidase (MPO) (a canonical neutrophil marker) (scale bar: 100 µm). Enlarged images showed the MPO‐positive neutrophils (black arrow). H) cf‐mtDNA levels in the BALF of septic mice were evaluated by RT‐qPCR (*n* = 8–10). Data represent mean ± SD; differences were compared by ANOVA with Tukey's multiple comparisons test. *p < 0.05, **p < 0.01, ***p < 0.001; ns, no significant difference.

As the cf‐mtDNA plays an important role in inflammatory responses and is contributed to the progression of sepsis‐associated ALI, it is anticipated that DNase‐I/HSA NMs, with the superior performances for degrading cf‐DNAs and prolonging lung retention, might be a potential therapeutic strategy for treating sepsis‐associated ALI. Next, DNase‐I/HSA NMs were tested for their performance in treating sepsis‐associated ALI. Considering that the cf‐mtDNA content in lung tissues did not change significantly at 3 h after LPS stimulation, but it increased significantly at 6 h (Figure S1B, Supporting Information). Therefore, we chose 3 h as the treatment time point. 3 h after LPS (10 mg kg^−1^) challenge in mice, the PBS, free DNase‐I (200 µg per mouse), or DNase‐I/HSA NMs (360 µg per mouse, equal to 200 µg of free DNase‐I) was given, respectively, via intratracheal administration (i.t). All experimental mice were euthanized at 24 h after LPS administration to collect the BALF and lung tissues (**Figure** **7**A, upper panel). Meanwhile, the survival rate was monitored every 12 h until 96 h after challenge with the lethal dose of LPS (30 mg kg^−1^) (Figure 7A, lower panel). It was found that over 40% septic mice died within 24 h when they were treated with PBS (Figure 7B). The free DNase‐I treatment had a slight better effect, especially in the early stage (within 24 h) after administration. Notably, the survival rate achieved 71.4% at 96 h after treatment with the DNase‐I/HSA NMs, while there was 42.9% in DNase‐I group and only 28.6% in LPS group. Moreover, the histopathological analysis of lung tissues showed that treatment with free DNase‐I moderately inhibited the LPS‐induced lung injury, whereas the administration of DNase‐I/HSA NMs exhibited obviously higher therapeutic efficiency, as indicated by the smallest lung injury score (Figure 7C,D). The severity of pulmonary edema in the pathogenesis of sepsis‐associated ALI was then evaluated by measuring the lung W/D weight ratio (Figure 7E). As expected, although free DNase‐I and DNase‐I/HSA NMs treatments were able to reduce pulmonary edema, DNase‐I/HSA NMs performed the greatest degree of remission (Figure 7E). Similarly, the analysis of LDH activity in BALF also revealed that DNase‐I/HSA NMs appeared to be more effective than free DNase‐I in ameliorating sepsis‐associated ALI (Figure 7F). Furthermore, the lung inflammation was estimated by measuring the cells infiltration and cytokines secretion in BALF. Consistently, both free DNase‐I and DNase‐I/HSA NMs significantly decreased the numbers of total inflammatory cells (Figure S6A, Supporting Information) and the levels of cytokines (Figure S6B–D, Supporting Information) in BALF, with DNase‐I/HSA NMs exerting a stronger inhibitory action. Since macrophages and neutrophils are the main immune cells that mediate lung inflammation during sepsis, we further detected the infiltration of macrophages and neutrophils in the lung tissues via immunohistochemical (IHC) analysis. In agreement with the previous results, there were abundant infiltration of macrophages (Figure S7, Supporting Information) and neutrophils (Figure 7G) in the lung tissue after LPS challenge, while free DNase‐I and DNase‐I/HSA NMs treatments effectively depressed the infiltration of immune cells, and the inhibition effect of DNase‐I/HSA NMs was more obvious. Finally, we found that both free DNase‐I and DNase‐I/HSA NMs could effectively clear increased cf‐mtDNA in the lungs during sepsis, and compared with free DNase‐I, DNase‐I/HSA NMs induced more significant cf‐mtDNA clearance (Figure 7H). This may be attributed to the self‐propulsion of DNase‐I/HSA NMs which contributed to prolong lung retention and increase catalytic efficiency, as mentioned above.^[^ ^38^, ^68^, ^69^ ^]^

Taken together, the DNase‐I/HSA NMs exhibited more effective to alleviate lung inflammation and protect mice from sepsis‐associated ALI, compare with free DNase‐I.

### 2.7 Biocompatibility of DNase‐I/HSA NMs

**Figure 8:**
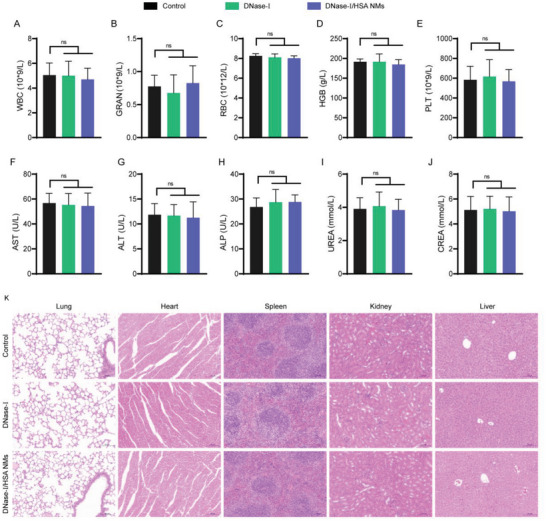
Toxicity of DNase‐I/HSA NMs in vivo. A–E) Blood panel data of normal mice (blank) and mice post either free DNase‐I or DNase‐I/HSA NMs administration at 24 h. F–J) Biochemical parameters analysis for liver function (ALT, AST, and ALP) and kidney function (BUN and CREA). K) Representative HE‐stained sections of main organs including lung, heart, spleen, kidney, and liver (scale bar: 100 µm). *n* = 4. Data represent mean ± SD; differences were compared by ANOVA with Tukey's multiple comparisons test. ns, no significant difference.

Biosafety, as the primary concern for the clinical translation of therapeutics, it is no exception for DNase‐I/HSA NMs. Two different cell lines, alveolar macrophages MH‐S and alveolar epithelial cells A549, were used to evaluate the potential cytotoxicity of DNase‐I/HSA NMs. CCK‐8 viability assay revealed that the DNase‐I/HSA NMs had no obvious cellular toxicity to either MH‐S cells or A549 cells within the investigation range of 1–800 µg mL^−1^ (Figure S8, Supporting Information). Furthermore, the potential in vivo toxicity of DNase‐I/HSA NMs was evaluated. The distribution of DNase‐I/HSA in major viscera (heart, spleen, kidney, and liver) was monitored by IVIS imaging 24 h post‐pulmonary administration. As shown in Figure S9 in the Supporting Information, both free DNase‐I and DNase‐I/HSA NMs mainly accumulated in the liver after pulmonary delivery, which might be attributed to the fact that liver is the main metabolic and detoxifying organ. In addition, the blood routine data for either free DNase‐I or DNase‐I/HSA NMs‐treated mice were all found to be normal (**Figure** **8**A–E and Figure S10, Supporting Information). The serum levels of AST, ALT, ALP, BUN, and CREA were also measured and all were found to be within normal limits, indicating that free DNase‐I and DNase‐I/HSA NMs did not induce any hepatic or renal injury (Figure 8F–J). And as observed in hematoxylin–eosin (HE) staining imaging, the main organs including lung, heart, spleen, kidney, and liver had not obvious histopathological changes in mice treated with either free DNase‐I or DNase‐I/HSA NMs (Figure 8K). Moreover, hemolysis assay showed that DNase‐I/HSA NMs exhibited superior hemocompatibility (Figure S11, Supporting Information). These results together highlighted the excellent biocompatibility of DNase‐I/HSA NMs, demonstrating that the application of DNase‐I/HSA NMs was feasible and ready for clinical practice.

## 3 Conclusion

In summary, we demonstrated that cf‐mtDNA was significantly increased in lung tissues during sepsis‐associated ALI, and played a vital role in activating alveolar macrophages and promoting pulmonary injury. Thus, targeting the clearance of accumulated cf‐mtDNA in the lungs could be a promising strategy for the management of sepsis‐associated ALI. Most importantly, we developed a novel DNase‐I‐powered NM system by crosslinking DNase‐I and HSA at an optimized ratio with glutaraldehyde as the crosslinker. The obtained DNase‐I/HSA NMs with well‐retained enzymatic activity exhibited enhanced diffusion at the pathological concentration of cf‐DNAs, which could enable their use in biomedical applications. Of note, owing to the self‐propulsive performance and appropriate particle size, the lung retention time of DNase‐I/HSA NMs was prolonged significantly. Moreover, pulmonary delivery of DNase‐I/HSA NMs exerted greater efficacy than free DNase‐I in treating sepsis‐associated ALI, which significantly improved survival rate and attenuated lung injury via efficient scavenging of cf‐mtDNA. Besides, the excellent biocompatibility of DNase‐I/HSA NMs was verified by in vitro cytotoxicity assay and in vivo safety evaluation. Overall, DNase‐I/HSA NMs are considered to be an attractive treatment strategy for treating sepsis‐associated ALI.

It is worth mentioning that cf‐DNAs are also markedly elevated in numerous diseases (e.g., inflammation, autoimmune, cancer, and cardiovascular disease) and play an important role in disease progression, indicating that our DNase‐I/HSA NMs could serve as a versatile platform for efficient treatment of multiple diseases. Due to its exceptional physiological stability, superior catalytic performance, excellent biocompatibility, and ease of preparation, such platform shows great potential for clinical translation in the future.

## 4 Experimental Section

### Materials

Recombinant DNase‐I was purchased from Coolaber Technology Co., Ltd. (Beijing, China). HSA was acquired from BoMei Biotechnology Co., Ltd. (Hefei, China). Cy5.5 *N*‐hydroxysuccinimide (NHS) ester was obtained from Yuanye Bio‐Technology Co., Ltd. (Shanghai, China). ICG‐NHS ester was acquired from Ruixi Biotech Co., Ltd. (Xian, China). Double‐stranded deoxyribonucleic acid (dsDNA) was purchased from Sigma‐Aldrich Co., Ltd. (Merck KGaA, Darmstadt, Germany). 4% paraformaldehyde and 4′,6‐diamidino‐2‐phenylindole (DAPI) were bought from Beyotime Biotechnology (Shanghai, China). The deionized water was prepared by the Milli‐Q water purification system (Millipore, MA, USA). All chemicals were of analytical‐reagent grade and were used without further purification.

### Synthesis of DNase‐I/HSA NMs

DNase‐I/HSA NMs were synthesized by using glutaraldehyde as crosslinking agent. Briefly, DNase‐I aqueous solution (1 mL, 5 mg mL^−1^) was mixed with HSA aqueous solution (1 mL, 5 mg mL^−1^). Then, 20 µL of 5% glutaraldehyde was added, and the resulting mixture was stirred overnight at room temperature. After that, the DNase‐I/HSA NMs were purified by dialysis against DPBS (Spectra/Por molecular‐porous membrane tubing 300 kDa MWCO, Spectrum, California, USA), condensed by ultrafiltration (Amicon Ultra 100 kDa MWCO, Millipore, Massachusetts, USA), and stored at 4 °C for future use.

DNase‐I NPs were constructed with the similar steps, except that only DNase‐I molecules were crosslinked via glutaraldehyde. Briefly, 10 µL of 5% glutaraldehyde was added to DNase‐I aqueous solution (2 mL, 5 mg mL^−1^), and the resulting mixture was stirred overnight at room temperature.

### Characterization of DNase‐I/HSA NMs

The morphology and structure of DNase‐I/HSA NMs were examined through biological TEM (HT7800, Hitachi, Tokyo, Japan) and SEM (Regulus 8100, Hitachi). The mean size and zeta potential of the particles were measured using a Zetasizer Nano ZSE (Malvern, Massachusetts, USA).

### Fluorescent Labeling of DNase‐I and DNase‐I/HSA NMs

NHS‐Cy5.5 or NHS‐ICG was used for labeling DNase‐I and DNase‐I/HSA NMs. Briefly, NHS‐Cy5.5 or NHS‐ICG was added to DNase‐I solutions and the reaction mixtures were stirred for 6 h at room temperature. After labeling reaction, the excess free dye was removed by dialysis against deionized water (Spectra/Por molecular‐porous membrane tubing 8 kDa MWCO, Spectrum). Labeled DNase‐I were then further condensed by ultrafiltration (Amicon Ultra 100, 10 kDa MWCO, Millipore) and stored at 4 °C. DNase‐I/HSA NMs were synthesized according to the method described previously.

### Fluorescence Spectrophotometric Assay

The proportion of DNase‐I in the form of DNase‐I/HSA NMs was determined by fluorescence spectrophotometry. Briefly, by comparing the fluorescence intensity of Cy5.5‐labeled DNase‐I/HSA NMs to Cy5.5‐labeled DNase‐I with known standard concentrations (as standard), the proportion of DNase‐I in the form of DNase‐I/HSA NMs could be quantified.

### Agarose Gel Electrophoresis

The enzymatic activity of DNase‐I was confirmed using a DNA digestion assay. Briefly, either free DNase‐I (2 µg and 20 µg) or DNase‐I/HSA NMs (2.5–100 µg) was mixed with 2 µg dsDNA in a final volume of 40 µL in DPBS. After reaction at 37 °C for 15 min, samples were electrophoresed on a 1% agarose gel and stained with YeaRed Nucleic Acid Gel Stain (Yeasen, Shanghai, China) for 10 min. DNA bands were visualized using the Tanon GIS system (Tanon, Shanghai China). The experiments were repeated at least three times.

### DNase‐I Catalytic Activity Assay

The enzymatic activity of DNase‐I was assessed using fluorometric DNase assay kit (Beyotime) in accordance with the manufacturer's instruction.

To evaluate the tolerance to pH, free DNase‐I and DNase‐I/HSA NMs were incubated in the buffers with different pHs for 2 h at room temperature.

To assess the resistance to proteinase K digestion, free DNase‐I and DNase‐I/HSA NMs were challenged with protease K (0.4 mg mL^−1^) for different time at room temperature.

### Animal Experiments

All animal experiments were performed in compliance with institutional guidelines and had been approved by the Institutional Animal Care and Use Committee of Southern Medical University (Protocol Registry Number: SMUL2022180).

### Animal Model Establishment and Treatment

To establish sepsis‐associated ALI model, the C57BL/6 mice (6–8 weeks old) were intraperitoneally (i.p) administered with LPS.

In order to explore the changes of cf‐DNAs content in the lung tissues during sepsis‐associated ALI, mice were sacrificed by dislocation at various time points following LPS challenge (10 mg kg^−1^), and BALF was collected for further analysis.

In order to clarify the role of cf‐DNAs in sepsis‐associated ALI, 1 h before LPS (10 mg kg^−1^) treatment, the mice were intratracheally instilled with PBS, cf‐nDNA (3 mg kg^−1^), or cf‐mtDNA (3 mg kg^−1^), respectively. The control group was treated with PBS. The animals were sacrificed at 24 h after LPS challenge.

For the assessment of treatment efficacy, 3 h after LPS (10 mg kg^−1^) administration, the mice were intratracheally instilled with PBS, free DNase‐I (200 µg per mouse), or DNase‐I/HSA NMs (360 µg per mouse, equal to 200 µg of free DNase‐I), respectively. The control group was treated with only PBS. The control group was treated with only PBS. The animals were sacrificed at 24 h after LPS challenge. For mortality, the mice were challenged with the lethal dose of LPS (30 mg kg^−1^), and then given the same treatment. The survival rate was monitored every 12 h until 96 h.

### Staining of cf‐DNAs

For detecting cf‐DNAs in lung tissues, frozen sections of mouse lungs were stained with 0.03 × 10^−6^ m SYTOX Green (Thermofisher Scientific, Waltham, USA) for 15 min and then washed with PBS three times, followed by DAPI incubation. Slides were imaged using confocal microscopy.

### cf‐DNAs Extraction and Quantification

cf‐DNAs were isolated from BALF using a QIAamp Circulating Nucleic Acid kit (Qiagen GmbH, Hilden, Germany), according to the manufacturer's instruction. cf‐DNAs concentration was measured with the Qubit dsDNA High Sensitivity assay kit (Yeasen, Shanghai, China).

### Histopathological Analysis

After fixation, the tissues were dehydrated in an ethanol gradient and xylene, embedded in paraffin, sliced into sections, and stained with HE. Morphological changes were observed using a photographic microscope (Leica Microscope Ltd., Wetzlar, Germany). Lung injury scores were evaluated by the Mikawa method,^[^ ^72^ ^]^ according to the following four targets which graded 0 to 4: alveolar congestion; pulmonary hemorrhage; neutrophils infiltration, and septal thickening, the summarization of the four variables represented the lung injury score.

### Lung W/D Weight Ratio

The harvested lung tissues were weighed immediately (wet weight) and reweighed following dehydration at 65 °C for 72 h (dry weight). Then, the ratio was calculated.

### BALF Analysis

The mice were sacrificed after LPS challenge and suspended on an endotracheal intubation platform. Then, the lungs were lavaged with 0.8 mL of ice‐cold PBS and withdrawn three times via tracheal catheter. BALF was collected and separated by centrifugation (1500 rpm, 10 min, 4 °C), and the supernatant was harvested for further analysis. Total protein and LDH activity in the supernatant were measured by bicinchoninic acid protein assay kit (Beyotime) and LDH cytotoxicity assay kit (Nanjing Jiancheng Bioengineering Institute, Nanjing, China), respectively, according to the manufacturer's instructions. In addition, the cell precipitate was collected and resuspended in 100 µL ice‐cold PBS and counted via a hemocytometer to obtain the total number of inflammatory cells.

### Determination of Inflammatory Cytokines Levels

The BALF levels of TNF‐α, IL‐1β, and IL‐6 were measured by corresponding ELISA kits, according to the manufacturer's instructions (Dakewe, Beijing, China).

### Determining Lung Retention

Following LPS challenge for 3 h, mice were intratracheally administered with either ICG‐labeled DNase‐I, DNase‐I NPs, or DNase‐I/HSA NMs, then sacrificed at various time points. The lung tissues were harvested and imaged ex vivo by an IVIS Spectrum imaging system (Spectral Instruments, Tucson, USA).

### Toxicity of DNase‐I/HSA NMs In Vivo

After intratracheal administration of PBS, free DNase‐I (200 µg per mouse), or DNase‐I/HSA NMs (360 µg per mouse, equal to 200 µg of free DNase‐I), respectively, for 24 h, mice were sacrificed and the main organs (lung, heart, liver, spleen, and kidney) as well as blood were harvested for further analysis. Briefly, histopathological examination was performed by HE staining, while hematological parameters and biochemical parameters were examined using an automatic hematology analyzer or an automatic biochemistry analyzer, respectively.

### IHC Staining

Lung sections were rehydrated and subjected to heat‐mediated antigen retrieval treatment, followed by treated with 3% H_2_O_2_ for 15 min, then blocked in 3% bovine serum albumin (BSA) for 30 min. Sections were incubated with rabbit anti‐MPO monoclonal antibody (dilution 1:1000, Abcam, Cambridge, USA) or rat anti‐F4/80 monoclonal antibody (dilution 1:200, Abcam) overnight at 4 °C. The following day, the sections were incubated with secondary antibodies conjugated with horseradish peroxidase (1:200, Beyotime) at room temperature for 1 h after repeated washing with PBS. After three times washing, the sections were then incubated with diaminobenzidine (Beyotime) within 5 min and counterstained with hematoxylin. The tissue sections were imaged using the Leica Microscope system.

### Cell Culture In Vitro

MH‐S cells (a mouse alveolar macrophage cell line) and A549 cells (a human alveolar epithelial cell line) were obtained from the Cell Bank of Typical Culture Collection of the Chinese Academy of Sciences (Shanghai, China). The MH‐S cells were cultured in RPMI medium 1640 (Gibco, New York, USA) supplemented with 10% FBS (Gibco) and 1% penicillin‐streptomycin (Gibco). The A549 cells were cultured in DMEM/F12 Medium (Gibco) supplemented with 10% FBS (Gibco) and 1% penicillin‐streptomycin (Gibco).

### Activation of Alveolar Macrophages

To examine the effects of BALF cf‐DNAs in regulating inflammation, BALF was separately collected from healthy mice and septic mice. After centrifugation, the supernatant was retained and treated with or without DNase‐I for 3 h at 37 °C. Subsequently, MH‐S cells were incubated with different samples of BALF for 6 h, and the activation state of macrophages was evaluated. The cells treated with only PBS were used as controls.

To further identify the activation effects elicited by cf‐DNAs in the macrophages, MH‐S cells were treated with either cf‐nDNA (5 µg mL^−1^) or cf‐mtDNA (5 µg mL^−1^) for 6 h, and the activation state of macrophages was evaluated. The cells treated with only PBS were used as controls.

### Alveolar Macrophage Uptake Quantification

To examine the cellular uptake of free DNase‐I and DNase‐I/HSA NMs in alveolar macrophage, MH‐S cells were seeded in 12‐well plates and allowed to adhere for 12 h. Then, MH‐S cells were incubated with Cy5.5‐labeled free DNase‐I or DNase‐I/HSA NMs for 6, 12, and 24 h, respectively. After incubation, cells were washed twice with PBS and stained with DAPI. The fluorescence images were acquired by inverted fluorescence microscope.

### Cytotoxicity of NMs

The cytotoxicity of DNase‐I/HSA NMs was measured by CCK‐8. Briefly, MH‐S cells and A549 cells were incubated with different concentrations of NMs (range from 0 to 800 µg mL^−1^) for 24 h, respectively. After that, the NMs were removed by washing with PBS. For CCK‐8 assay, the CCK8 detection kit (Beyotime) was used according to the manufacturer's protocol.

### Immunofluorescence Assay

After challenge, MH‐S cells were fixed with 4% paraformaldehyde and permeabilized with 0.3% Triton X‐100. Afterward, the cells were blocked with 5% BSA, and stained overnight with primary antibodies (1:200) at 4 °C. The following day, the cells were incubated with fluorescent secondary antibodies (1:200) at room temperature for 1 h. After incubation, cells were washed with PBS and stained with DAPI. The images were acquired using confocal microscopy.

### Real‐Time Quantitative PCR

Quantification of cf‐nDNA and cf‐mtDNA in cf‐DNAs extracted from BALF was performed by SYBR Green‐based real‐time quantitative PCR (RT‐qPCR) as described previously.^[^ ^73^ ^]^ Briefly, the RT‐qPCR was performed using Hieff UNICON qPCR SYBR Green Master Mix (Yeasen) with primer pairs listed in Table S1 in the Supporting Information to evaluate the content of cf‐nDNA and cf‐mtDNA by amplifying the GAPDH gene standing for nDNA and ND1 gene representing mtDNA. Then, the copy number of cf‐mtDNA or cf‐nDNA was calculated from the linearity constructed by dosage‐dependent standard plasmid DNA solutions. RT‐qPCR was performed using the LightCycler 480 Real‐Time PCR System (Roche Applied Science, Indianapolis, IN) according to the following programme: initial denaturation at 95 °C for 30 s, 40 cycles of denaturation at 95 °C for 15 s, and annealing/extension at 60 °C for 30 s. The amplification curve and melting curve were examined to evaluate specific amplification of standard plasmid DNA (Figures S12 and S13, Supporting Information).

To examine the mRNA levels of proinflammatory cytokines in MH‐S cells, RT‐qPCR was performed as previously described.^[^ ^74^ ^]^ Briefly, total RNA was extracted with the Cell Total RNA Isolation kit (Foregene, Chengdu, China), and cDNA was prepared using a Reverse Transcription Kit (Yeasen). Then, RT‐qPCR was performed using Hieff UNICON qPCR SYBR Green Master Mix (Yeasen) with primer pairs listed in Table S1 in the Supporting Information. RT‐qPCR was performed using the LightCycler 480 Real‐Time PCR System (Roche Applied Science) according to the following programme: initial denaturation at 95 °C for 30 s, 40 cycles of denaturation at 95 °C for 15 s, and annealing/extension at 60 °C for 30 s. The expression level of β‐actin was used as an internal control and the relative expression of proinflammatory cytokines was quantified with the 2^−ΔΔCT^ method.

### Extraction and Fragmentation of nDNA and mtDNA

To examine the potential roles of nDNA and mtDNA in sepsis‐associated ALI, nDNA and mtDNA were extracted from mouse livers using the methods previously described.^[^ ^45^ ^]^ Briefly, nuclear and mitochondrial fractions were purified from the livers of C57BL/6 mice subjected to ischemia and reperfusion with the Nuclear Extraction Kit (Solarbio, Beijing) and the Mitochondria Isolation Kit (Beyotime), respectively, according to the manufacturer's instructions. The nuclei were used to isolate nDNA with the QIAamp DNA Blood Mini Kit (Qiagen GmbH), whereas mitochondria were used to extract mtDNA. The obtained DNA was sheared to 150–200 bp fragments using an ultra sonicator system to mimic cf‐DNAs size. The DNA concentrations and purity were analyzed using a NanoDrop 2000 instrument (Thermo Fisher Scientific, Waltham, MA, USA).

### Hemolysis Assay

2% suspension of red blood cells was incubated with various concentrations (50, 100, 400, and 800 µg mL^−1^) of DNase‐I/HSA NMs at 37 °C for 2 h. Afterward, samples were centrifuged and the supernatant was analyzed by spectrophotometric tests at 570 nm. 1% TritonX‐100 and PBS were used as positive and negative controls, respectively.

### Movement Recording and Analysis

Nikon Ti2‐A inverted optical microscope equipped with a high‐speed camera (sCMOS) and NIS Elements AR3.2 software was used to record the motion behavior of DNase‐I/HSA NMs. The motion trajectories of DNase‐I/HSA NMs were recorded for 20 s under different dsDNA concentrations (0 × 10^−6^, 0.025 × 10^−6^, 0.05 × 10^−6^, 0.15 × 10^−6^, and 1.5 × 10^−6^ m). Subsequently, the tracking image and the speed of DNase‐I/HSA NMs were analyzed by ImageJ plugin manual tracking. MSD analysis was conducted by using the self‐diffusiophoretic model.

### Statistical Analysis

The experimental data were expressed as mean ± standard deviation (SD) and were analyzed with GraphPad Prism 8.0 (GraphPad Software Inc., La Jolla, CA, USA). Student's *t*‐test was used to compare the differences between two groups, and multiple comparisons were conducted using one‐way ANOVA with Tukey's multiple comparisons test. *p < 0.05, **p < 0.01, and ***p < 0.001 were applied to annotate statistical significance.

## Conflict of Interest

The authors declare no conflict of interest.

## Supporting information

Supporting Information

Supplemental Movie 1

Supplemental Movie 2

Supplemental Movie 3

Supplemental Movie 4

Supplemental Movie 5

## Data Availability

The data that support the findings of this study are available from the corresponding author upon reasonable request.
