# Supplementary material for: Self‐Propelled Proteomotors with Active Cell‐Free mtDNA Clearance for Enhanced Therapy of Sepsis‐Associated Acute Lung Injury
Source: Adv Sci (Weinh). 2023 Jul 30;10(27):2301635. doi: 10.1002/advs.202301635 (PMC10520684; doi:10.1002/advs.202301635)
Supplement: Supplementary file 1 — Supporting Information [file ADVS-10-2301635-s001.pdf]

ADVANCED SCIENCE
Open Access

Supporting Information

for *Adv. Sci.*, DOI 10.1002/advs.202301635

Self-Propelled Proteomotors with Active Cell-Free mtDNA Clearance for Enhanced Therapy of Sepsis-Associated Acute Lung Injury

Weichang Huang, Lihong Wen, Hao Tian, Jiamiao Jiang, Meihuan Liu, Yicheng Ye, Junbin Gao, Ruotian Zhang, Fei Wang, Huaan Li, Lihan Shen, Fei Peng* and Yingfeng Tu**

Supporting Information

Self-propelled Proteomotors with active Cell-free mtDNA Clearance for Enhanced Therapy of Sepsis-associated Acute Lung Injury

Weichang Huang, Lihong Wen, Hao Tian, Jiamiao Jiang, Meihuan Liu, Yicheng Ye, Junbin Gao, Fei Wang, Ruotian Zhang, Huaan Li, Lihan Shen, Fei Peng*, Yingfeng Tu**

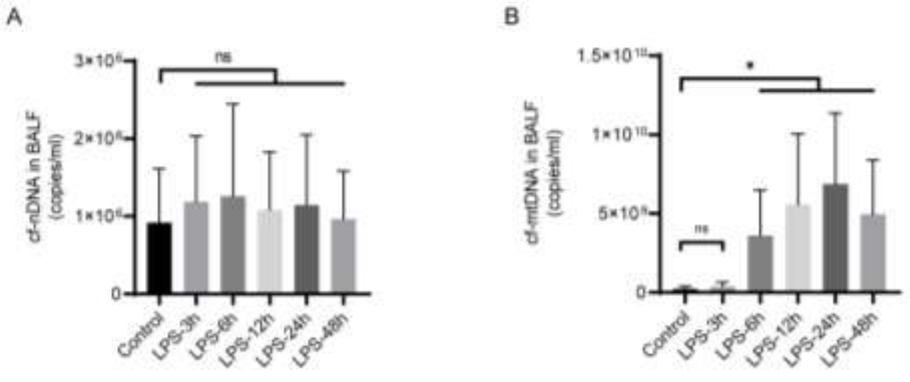

Figure S1. Dynamic changes of cf-mtDNA and cf-nDNA BALF levels during sepsis-associated ALI. A, B, Levels of cf-mtDNA (A) and cf-mtDNA (B) in the BALF of septic mice were evaluated by RT-qPCR. n = 8-12. Data represent mean ± SD; differences were compared by one-way analysis of variance (ANOVA) with Tukey's multiple comparisons test. *$p < 0.05$; ns, no significant difference.

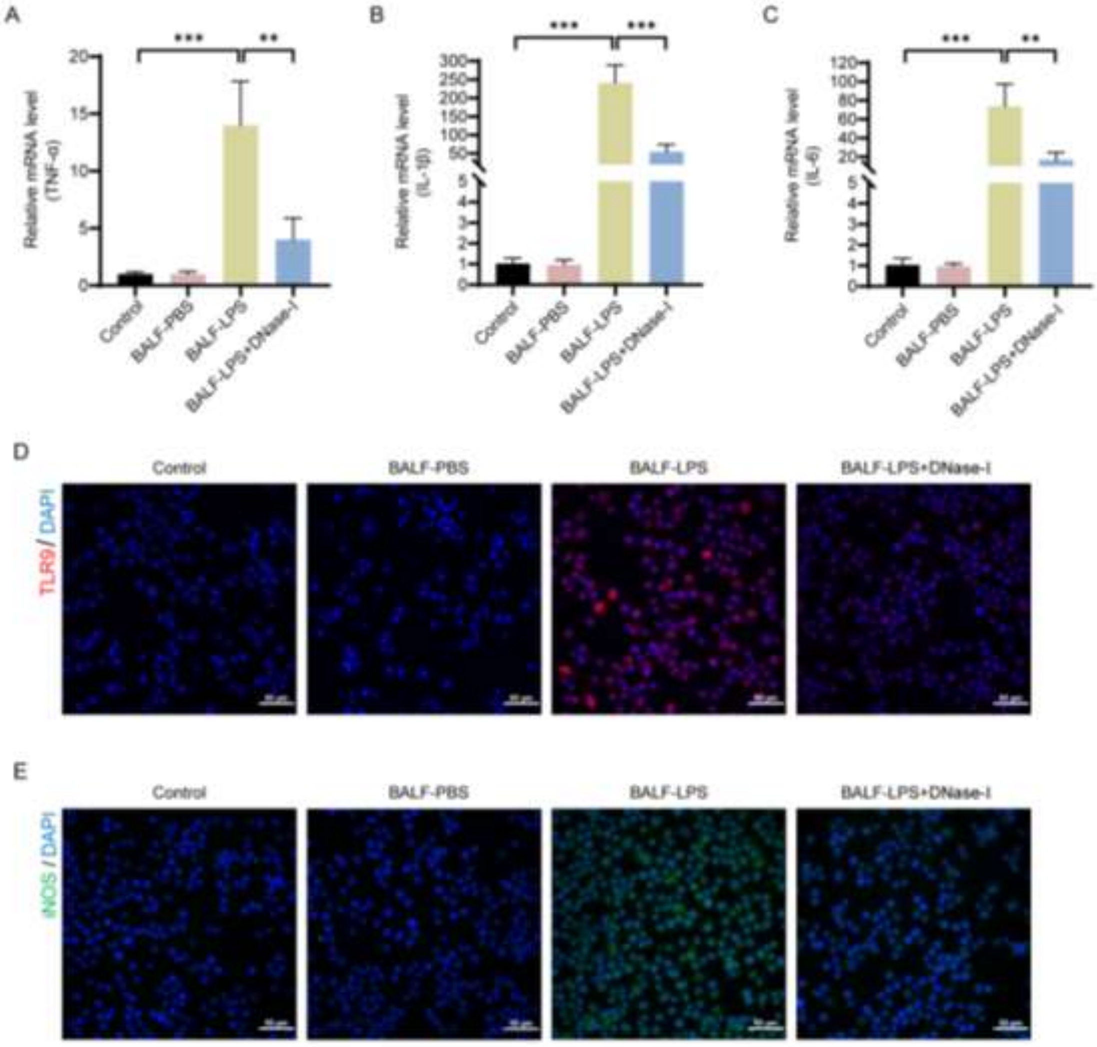

Figure S2. The activation status of alveolar macrophages following co-incubation with BALF. Alveolar macrophage cells were activated by BALF obtained from septic mice, which was significantly attenuated by DNase-I treatment. A-C, mRNA levels of TNF-a (A), IL-1β (B) and IL-6 (C) in alveolar macrophage cells (MH-S) after incubating with PBS or BALF for 6 h were evaluated by RT-qPCR. n = 3. E, F, The expressions of TLR9 (D) and iNOS (E) in alveolar macrophage cells were assessed by fluorescence staining (Scale bar: 50 μm). All cell experiments were performed at least in triplicate. The corresponding groups respectively were: Control (co-incubation with PBS), BALF-PBS (co-incubation with BALF obtained from PBS-challenged mice), BALF-LPS (co-incubation with BALF obtained from LPS-challenged mice), BALF-LPS+DNase-I (co-incubation with BALF which obtained from LPS-challenged mice and treated with DNase-I for 3 h). Data represent mean ± SD; differences were compared by ANOVA with Tukey's multiple comparisons test. *$p < 0.05$, **$p < 0.01$, ***$p < 0.001$.

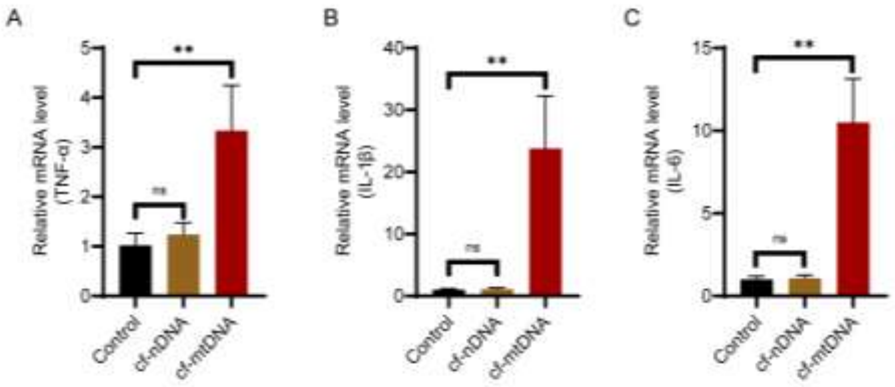

Figure S3. The mRNA levels of pro-inflammatory cytokines in alveolar macrophages following cf-nDNA and cf-mtDNA challenge. The mRNA levels of pro-inflammatory cytokines were markedly increased in alveolar macrophage cells after treating with cf-mtDNA for 6 h. A-D, mRNA levels of TNF-α (A), IL-1β (B) and IL-6 (C) in alveolar macrophage cells (MH-S) challenged with PBS, cf-nDNA or cf-mtDNA for 6h were evaluated by RT-qPCR. n = 3. The corresponding groups respectively were: Control (treatment with PBS), cf-nDNA (treatment with cf-nDNA), cf-mtDNA (treatment with cf-mtDNA). Data represent mean ± SD; differences were compared by ANOVA with Tukey's multiple comparisons test. *$p < 0.05$, **$p < 0.01$; ns, no significant difference.

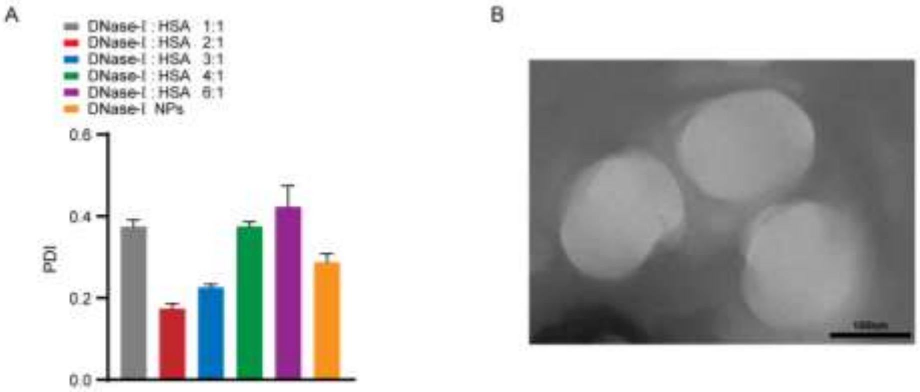

Figure S4. Physicochemical characterization of DNase-I/HSA NMs. A, The PDI of DNase-I/HSA NMs synthesized with different molar ratios between DNase-I and HSA. n = 3. B, TEM image of DNase-I/HSA NMs (Scale bar: 100 nm). All experiments were performed at least in triplicate. Data represent mean ± SD.

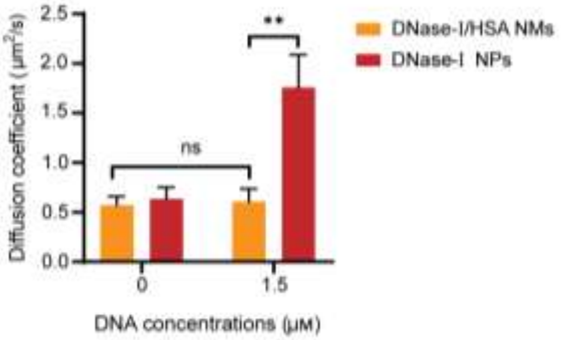

Figure S5. The motility performance of DNase-I NPs and DNase-I/HSA NMs. The diffusion coefficient of DNase-I NPs and DNase-I/HSA NMs in the presence of 1.5 μM dsDNA were measured by DLS. n = 6. Data represent mean ± SD; differences were compared by ANOVA with Tukey’s multiple comparisons test. *$p < 0.05$, **$p < 0.01$; ns, no significant difference.

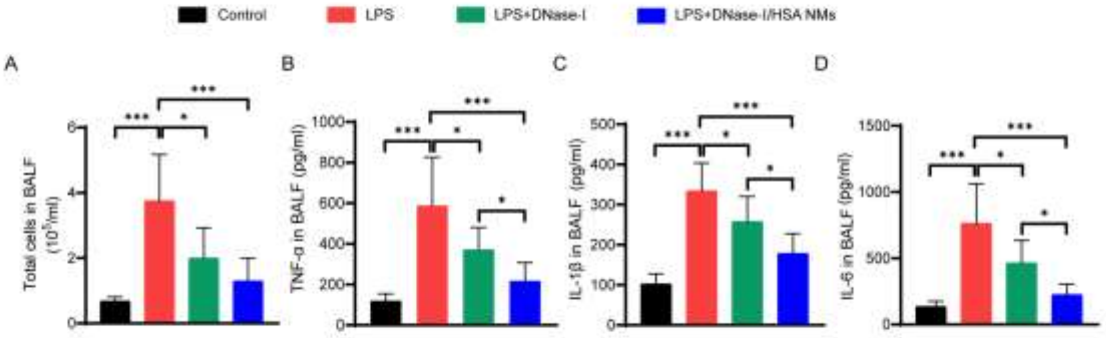

Figure S6. Assessment of lung inflammation. The lung inflammation was estimated by measuring the cells infiltration and cytokines secretion in BALF. A, Total number of inflammatory cells in BALF were counted via a haemocytometer (n = 6). B-D, The concentrations of inflammatory cytokines TNF-α (B), IL-1β (C) and IL-6 (D) in BALF were determined by ELISA (n = 8). Data represent mean ± SD; differences were compared by ANOVA with Tukey's multiple comparisons test. **p* < 0.05, ***p* < 0.01, ****p* < 0.001.

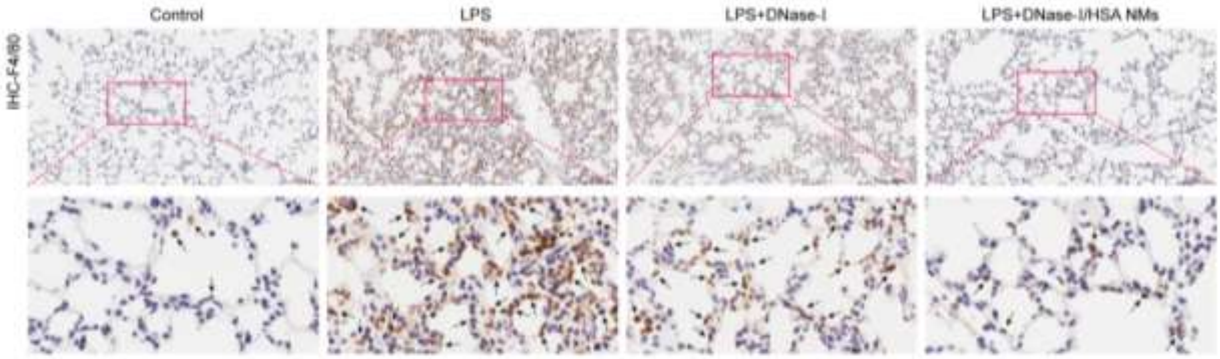

Figure S7. Assessment of macrophage infiltration. Pulmonary macrophage infiltration was detected by IHC for F4/80 (a pan macrophage marker) (Scale bar: 100 μm). Enlarged images showed the F4/80-positive macrophages (Black arrow).

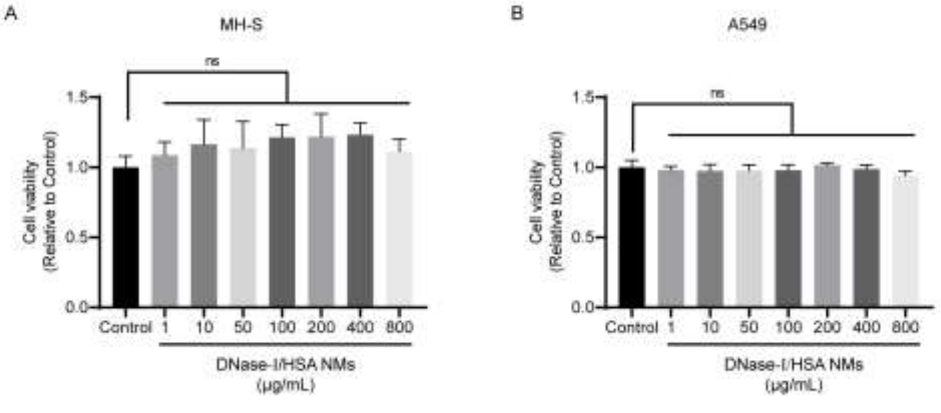

Figure S8. Cytotoxicity of DNase-I/HSA NMs. A, Cell viability of alveolar macrophages MH-S treated with various concentrations of DNase-I/HSA NMs for 24 h. B, Cell viability of alveolar epithelial cells A549 treated with various concentrations of DNase-I/HSA NMs for 24 h. n = 6. Data represent mean ± SD; differences were compared by ANOVA with Tukey's multiple comparisons test. ns, no significant difference.

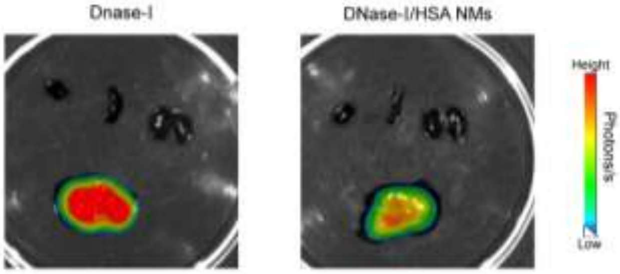

Figure S9. The biodistribution of DNase-I/HSA in ALI models. The biodistributions of free DNase-I and DNase-I/HSA NMs in internal organs (heart, spleen, kidney and liver) 24 h after administration were monitored by IVIS imaging.

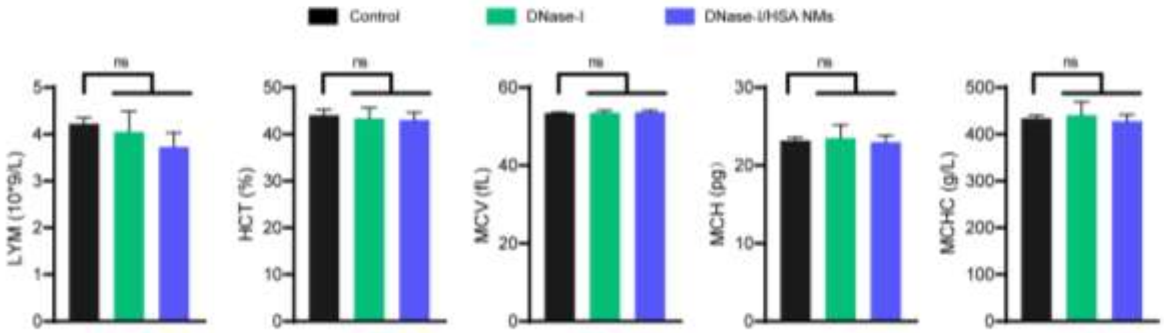

Figure S10. Blood panel data for *in vivo* safety evaluation. Blood panel data of normal mice (blank) and mice post either free DNase-I or DNase-I/HSA NMs administration at 24 h. n = 4 mice/group. Data represent mean ± SD; differences were compared by ANOVA with Tukey's multiple comparisons test. ns, no significant difference.

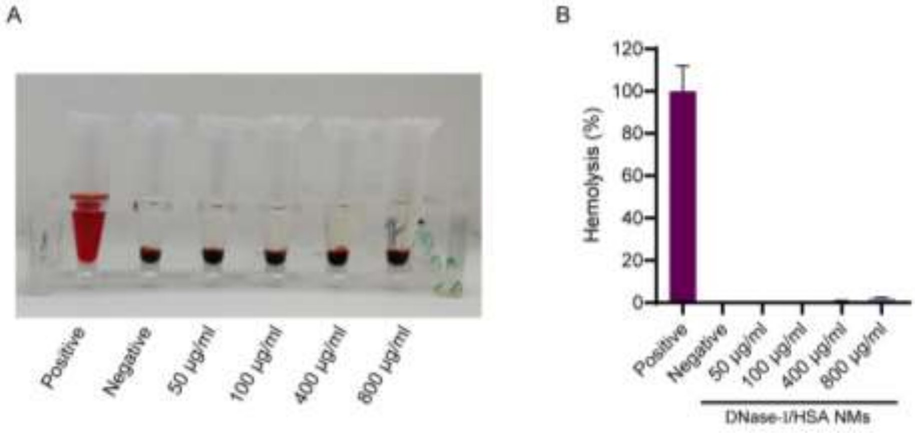

Figure S11. Hemolysis assay of DNase-I/HSA NMs. (A) Photographs and (B) hemolysis assay of negative, positive and DNase-I/HSA NMs with different concentrations. n = 3. Data represent mean ± SD.

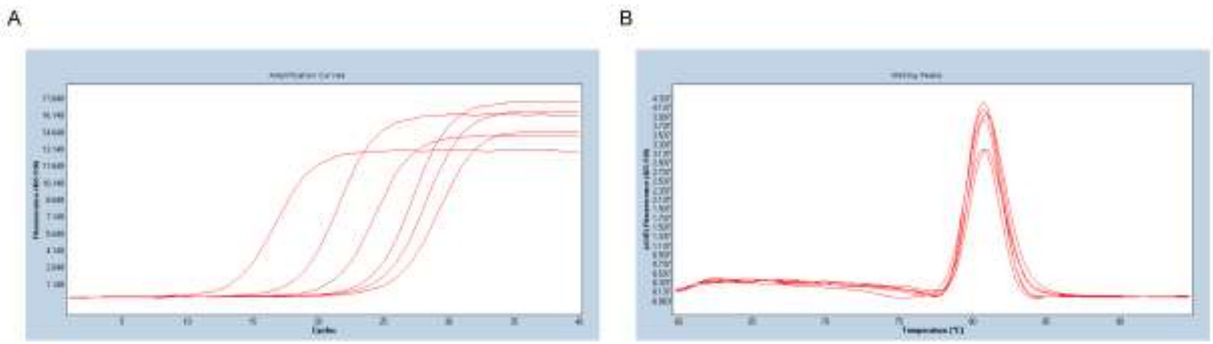

Figure S12. Assessment for the specific amplification of GAPDH plasmid DNA. The specific amplification of GAPDH plasmid DNA (for nDNA), was evaluated by amplification curve (A) and melting curve (B).

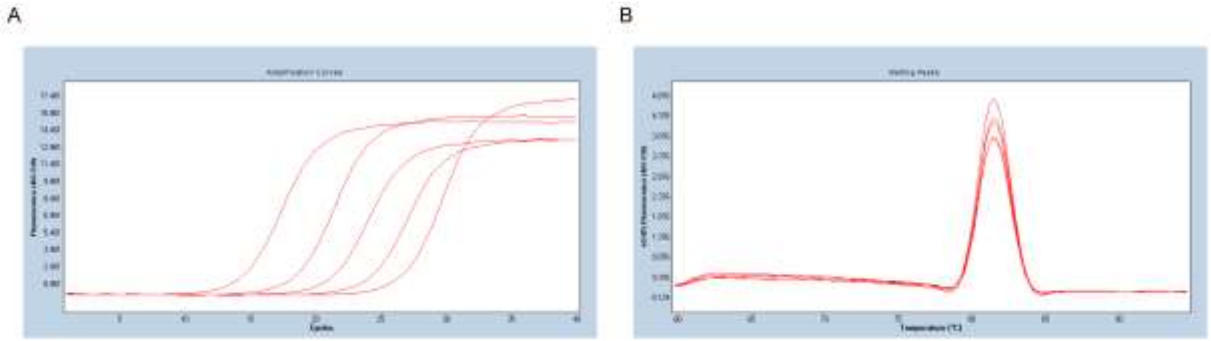

Figure S13. Assessment for the specific amplification of ND1 plasmid DNA. The specific amplification of ND1 plasmid DNA (for nDNA), was evaluated by amplification curve (A) and melting curve (B).

Table S1. List of primers used for RT-qPCR

Gene	Forward Primer	Reverse Primer
GAPDH	CTCATGGTATGTAGGCAGTGG	ACAAAGAAGGGTCCACTCATGG
mt ND1	CTAGCAGAAACAAACCGGGC	CCGGCTGCGTATTCTACGTT
TNF-a	ACTGAACTTCGGGGTGATCGGT	TGGTTTGCTACGACGTGGGCTA
IL-1β	TTGACGGACCCCAAAAGATG	CAGCTTCTCCACAGCCACAA
IL-6	CCAGAAACCGCTATGAAGTTCC	CGGACTTGTGAAGTAGGGAAGG
β-actin	GAAATCGTGCGTGACATCAAAG	TGTAGTTTCATGGATGCCACAG

Supplementary Movies:

Movie S1. Brownian motion of DNase-I/HSA NMs in aqueous solution.

Movie S2. Enhanced diffusion of DNase-I/HSA NMs in 0.025 μM DNA solution.

Movie S3. Enhanced diffusion of DNase-I/HSA NMs in 0.05 μM DNA solution.

Movie S4. Enhanced diffusion of DNase-I/HSA NMs in 0.15 μM DNA solution.

Movie S5. Enhanced diffusion of DNase-I/HSA NMs in 1.5 μM DNA solution.
